# Improvements with burosumab treatment in an early access programme for adults with X-linked hypophosphataemia: A case series of three patients

**DOI:** 10.1016/j.bonr.2024.101814

**Published:** 2024-11-12

**Authors:** Julia Day, Chandrin Jayatilleke, Matthew Roy

**Affiliations:** a Department of Rheumatology, Bristol Royal Infirmary, Bristol, BS2 8HW, UK; b Department of Rheumatology, Royal United Hospital, Bath BA1 3NG, UK

**Keywords:** Burosumab, Patient-reported outcome, Case series, Early access, X-linked hypophosphatemia

## Abstract

X-linked hypophosphataemia (XLH) is a life-long phosphate-wasting disorder that causes skeletal deformities, pain, stiffness, and fatigue and impairs quality of life. Burosumab was approved for use in adults in 2020. We describe three adults with persistent XLH symptoms who received burosumab treatment in a real-world setting. Patients report improvements in pain, mobility, physical function, energy, fatigue, and mental wellbeing through patient-reported outcome measures, enriched with further detail from written testimonials.

## 1 Introduction

X-linked hypophosphataemia (XLH) is a rare, genetic, life-long, phosphate-wasting disorder caused by loss-of-function mutations in the *PHEX* (phosphate-regulating endopeptidase homologue, X-linked) gene. The resulting excess circulating levels of fibroblast growth factor 23 (FGF23) lead to renal phosphate wasting and hypophosphataemia (Beck-Nielsen et al., 2019), with deleterious effects on the mineralization, growth, and quality of bones, abnormalities in muscle structure and function, and impaired dental mineralization (Beck-Nielsen et al., 2019; Chen et al., 2018; Orlando et al., 2022).

XLH usually appears in early childhood and is characterized by rickets, skeletal deformities, short stature, and dental abscesses (Carpenter et al., 2011; Linglart et al., 2014). By adulthood, dominant musculoskeletal features include fractures, enthesopathies, osteoarthritis with osteophytes, and spinal stenosis (Javaid et al., 2022; Skrinar et al., 2019a); features accumulate with age (Javaid et al., 2022). Adults with XLH self-report bone and joint pain, stiffness, and fatigue, as well as impairments in physical function, sleep, mental wellbeing, and health-related quality of life (HRQL) (Che et al., 2016; Cole et al., 2023; Javaid et al., 2022; Lo et al., 2020; Skrinar et al., 2019a).

Current guidelines recommend that adults with symptomatic XLH are treated with oral phosphate supplementation and active vitamin D to reduce osteomalacia and improve dental health (Haffner et al., 2019). This has been conventional therapy for the past four decades but it stimulates FGF23 levels and renal phosphate wasting, and is associated with adverse effects such as hyperparathyroidism and nephrocalcinosis (Haffner et al., 2019). In addition, patients need to take multiple daily doses of medication and undergo frequent blood tests and dose titration (Haffner et al., 2019).

Burosumab is a fully human monoclonal antibody against FGF23. Inhibition of FGF23 by burosumab results in increased reabsorption of phosphate by the kidneys, restoring serum phosphate concentrations to within the normal range (Insogna et al., 2018). The efficacy and safety of burosumab in adults have been demonstrated in a phase 3 randomized controlled study (NCT02526160): serum phosphate concentrations increased to within the normal range, with concomitant improvements after 24 weeks' treatment in stiffness and physical function, measured using the Western Ontario and McMaster Universities Osteoarthritis Index (WOMAC®), and worst pain, measured using the Brief Pain Inventory short-form (BPI-SF) (Insogna et al., 2018). Continued benefit with burosumab has been demonstrated at 48 and 96 weeks (Briot et al., 2021; Insogna et al., 2018) and for a further 48 weeks in an open-label phase 3b study (Kamenicky et al., 2023). Burosumab is approved in Europe for the treatment of XLH in adults and in children aged 1–17 years with radiographic evidence of bone disease (EMA, 2022). Since March 2023, adults in Scotland can be prescribed burosumab via the ultra-orphan pathway, pending reassessment in 3 years' time (SMC, 2023). The National Institute for Health and Care Excellence published final draft guidance in July 2024 recommending use of burosumab within the National Health Service for the treatment of adults with XLH (NICE, 2024).

Recognizing the unmet need for an effective treatment to restore phosphate homeostasis and improve symptoms, and with potential to slow the progression of the clinical manifestations of XLH, burosumab was made available to patients via an early access programme (EAP), initiated in 2019, for those aged ≥18 years with a confirmed diagnosis of XLH based on family history or confirmed *PHEX* mutation and persistent symptoms despite treatment with conventional therapy. Three subgroups of adults with XLH were eligible for burosumab treatment via the EAP: those with non-healing or slow-healing fractures or pseudofractures; those with moderate or severe pain, stiffness, and/or fatigue attributable to XLH and that impacts HRQL; and those who have undergone or require orthopaedic surgery, dental surgery with bone involvement, or spinal surgery, for a predefined period before and after surgery.

The treatment of adults with XLH via the EAP provides a valuable opportunity to collect evidence relating to its use in real-world clinical practice, supplementing evidence from clinical trials. We present a case series of three adults with XLH who received treatment with burosumab via the EAP, including laboratory results, patient-reported outcomes (PROs), and patient testimonials. Together these provide a unique perspective on XLH-related impairment when receiving conventional therapy and the impact of burosumab treatment.

## 2 Case studies

Patients were recruited as part of the EAP at a single centre in Bristol (UK). All had a confirmed diagnosis of XLH and met the eligibility criteria for the EAP programme: age ≥ 18 years; confirmed diagnosis of XLH based on familial history or confirmed *PHEX* mutation; presence of debilitating symptoms including (but not limited to) pain, stiffness, and fatigue. As part of the EAP, patients completed the BPI-SF, WOMAC, and the EuroQol five-dimension, five-level health status questionnaire (EQ-5D-5L). Patients were invited to provide a written response outlining their personal experience of burosumab and the impact it had on their health. The three cases presented in this case series were selected because they had completed at least one PRO questionnaire before and after burosumab treatment and provided a testimonial.

Written informed consent to disseminate this information was obtained from patients by the treating physician.

### 2.1 Clinical background

The case studies (CS) comprised three adults (two female, one male), all of whom were White British had the XLH diagnosis confirmed by *PHEX* mutation testing.

- CS1 was diagnosed with XLH at age 9 years after presenting with knee pain and hypophosphataemic rickets. The patient had knee pain throughout their life and had developed marked deformity and medial collapse of the knee joints when referred to orthopaedics at age 30 years. She subsequently developed severe osteoarthritis in the knees and a tibial stress fracture at age 57 years.
- CS2 was diagnosed with XLH in infancy based on low serum phosphate, stiffness, limited range of movement in key joints, and a family history of XLH. On referral to the current specialist, he had widespread joint pain, stiffness and fatigue, low mood, tinnitus, and hearing loss, but did not have any orthopaedic history.
- CS3 was also diagnosed with XLH at a young age based on low serum phosphate concentrations, symptoms, and a family history. Her history at referral included significant joint pain, stiffness, and fatigue and she had developed spinal stenosis. She had undergone orthopaedic surgery for leg straightening at age 6 years and subsequent surgery for a femur fracture, requiring multiple revisions.

All three patients had poor dental health and reported abscesses and loss of teeth. All three reported that they had given up work because of symptoms.

All three patients received treatment with oral phosphate supplements and active vitamin D during childhood. Only CS1 reported restarting conventional therapy, at age 40+ years.

### 2.2 Burosumab treatment

All three patients started burosumab at a dose of 1 mg/kg every 4 weeks and did not receive any concurrent treatment for XLH. CS1 stopped conventional therapy when starting burosumab as per the guidance in the Summary of Product Characteristics (EMA, 2022); CS2 and CS3 were not taking conventional therapy at the time of referral. All patients had normal renal function at baseline (before starting burosumab treatment).

### 2.3 Laboratory findings

All three patients had low serum phosphate concentrations before burosumab treatment (Table 1). Serum phosphate concentration was normalized at 3 months in CS1 and maintained within the normal range by 12 months. CS2 had a level just below the normal range (0.79 mmol/L) at 12 months but no further values were reported. CS3 had a level close to normal (0.75 mmol/L) at 12 months, when the dose of burosumab was increased to 60 mg; serum phosphate concentration reached the normal range (0.98 mmol/L) within 24 months of first starting burosumab.

**Table 1:** Laboratory findings before and during burosumab treatment, where reported.

Laboratory value (normal range)	Timing	CS1	CS2	CS3
Fasting serum phosphate (0.80–1.50 mmol/L)	Before BT	0.43	0.39	0.60
	During BT	0.96 (3 mo) 0.95 (12 mo)	0.79 (1 mo)	0.75 (12 mo) 0.98 (24 mo)
25-hydroxyvitamin D (>50 nmol/L)	Before BT	Normal	38	NR
	During BT	59	NR	NR
Serum calcium (2.1–2.6 mmol/L)^a^	Before BT	Normal	Normal	Normal
	During BT	2.52 (3 mo) 2.55 (12 mo)	2.42 (1 mo) 2.65 (4 mo)	NR
PTH (1.6–6.9 pmol/L)	Before BT	8.8	12.9	9.5
	During BT^b^	7.5 (12 mo)	NR	NR
ALP (30–130 U/L)	Before BT	180	NR	170
	During BT	168 (12 mo)	194 (1 mo) 136 (4 mo)	NR

ALP, alkaline phosphatase; BT, burosumab treatment; mo, month(s); CS, case study; NR, not reported; PTH, parathyroid hormone.

a Cross-linked C-telopeptide of type I collagen (CTX) bone turnover marker elevated at 1.29 μg/L (0.1–0.5) indicating increased bone resorption.

All three patients had elevated parathyroid hormone before starting burosumab treatment but levels were nearer normal after 12 months of treatment in CS1 (levels not reported for CS2 and CS3). Alkaline phosphatase levels were elevated before treatment in CS1 and CS3 and decreased during treatment (where reported). Vitamin D (25-hydroxyvitamin D) levels were normal in CS1 but below normal in CS2 (not reported for CS3). All three patients had normal serum calcium levels before starting burosumab treatment. Serum calcium levels were at the top end of the normal range during treatment.

Burosumab was well tolerated: none of the patients reported any side-effects or adverse events. No issues with treatment adherence were reported.

### 2.4 Patient-reported symptoms and outcomes

#### 2.4.1 Before burosumab

Patients reported high levels of pain before taking burosumab: WOMAC Pain scores were 15, 17, and 13 (out of 20) and BPI-SF Worst Pain scores were 5, 9, and 8 (out of 10) (Table 2). These high levels of pain are supported by the testimonials: patients reported being in ‘constant pain’ (CS1) and being in pain ‘most of my life’ (CS3). One patient reported a devastating impact of pain on their mental wellbeing: ‘… my mental health was bad and I was often feeling depressed, anxious, and having suicidal thoughts from being in so much pain all the time’ (CS2).

**Table 2:** Patient-reported outcome scores.

PRO measure	Score	Score range and interpretation	CS	Score before burosumab	Score on burosumab^a^	Change^b^
BPI-SF	Worst Pain	0–10 (increasing severity)	1	5	3	**2 ↑**
			2	9	4	**5 ↑**
			3	8	6	**2 ↑**
WOMAC	Stiffness	0–8 (increasing severity)	1	8	4	**4 ↑**
			2	7	4	**3 ↑**
			3	6	2	**4 ↑**
	Pain	0–20 (increasing severity)	1	15	12	**3 ↑**
			2	17	Not reported	
			3	13	15	2 ↓
EQ-5D-5L	Index score^c^	0–1 (1 = perfect health)	1	0.277	0.790	0.513 ↑
			2	0.121	0.725	0.604 ↑
			3	0.699	0.720	0.021 ↑
	EQ-VAS	0–100 % (poorest to perfect health)	1	40	75	35 ↑
			2	25	72	50 ↑
			3	65	65	0

BPI-SF, Brief Pain Inventory Short-form; CS, case study; EQ-5D-5L, EuroQol five-dimension, five-level health status questionnaire; EQ-VAS, EuroQol visual analogue scale; PRO, patient-reported outcome; WOMAC, Western Ontario and McMaster Universities Osteoarthritis Index.

a 12 months after starting burosumab (CS1, CS2), 6 months for CS3.

b ↑ indicates an improvement; ↓ indicates worsening; **bold** indicates clinically relevant improvements for the BPI-SF and WOMAC (based on responder thresholds: 1.72 for BPI-SF; ≥1 for WOMAC Stiffness; 2.2 for WOMAC Pain) (Skrinar et al., 2019b; Skrinar et al., 2019c); XLH-specific thresholds are not available for the EQ-5D-5L.

c Index scores for EQ-5D-5L based on the weighted formula for the UK population.

Similarly, high levels of stiffness were reported on the WOMAC Stiffness scale (scores of 8, 7, and 6 out of 8) (Table 2) and were supported by the patient testimonials: ‘Most of my life before burosumab I have … suffered from stiffness’ (CS3).

In their testimonials, patients also describe debilitating mobility problems: ‘… every step felt like I was wading, unbalanced through water … My legs felt as if they could not support my weight, my mobility was limited …’ (CS1); ‘… before it would take up to an hour before I could stand up and walk properly … my balance was always off and I'd feel unsteady on my feet when standing or walking and would often trip up over my own feet’ (CS2). Patients needed physical support from other people or walking aids: ‘… It would be a struggle to get up off the ground and I would definitely need assistance from a person or aid like a stick or using the side’ (CS2). Broader problems with physical function were also described: ‘I would really struggle to crouch or bend over before my treatment’ (CS2). Physical symptoms compromised patients' ability to perform normal daily activities: ‘I carried out household tasks sat on a stool with wheels, as I was unable to stand for long periods’ (CS1); ‘Every day had to be carefully planned so I could cope with just normal activities’ (CS3).

Tiredness and fatigue were a common theme: ‘I was very tired and fatigued all the time’ (CS2); ‘Most of my life … I have been … very fatigued’. (CS3).

HRQL scores (EQ-5D-5L index and visual analogue scale [EQ-VAS] scores) were low for two patients (Table 2): index scores for CS1 and CS2 were 0.228 and 0.121 (range 0–1); and EQ-VAS scores were 40 and 25 (range 0–100). Scores for CS3 were 0.699 and 65, respectively.

#### 2.4.2 During burosumab

Patients' BPI-SF Worst Pain scores had improved 6–12 months after starting burosumab (improvements of 2, 5, and 2 points out of 10), which exceeds the XLH-specific clinically meaningful threshold of a 1.72 point improvement (Skrinar et al., 2019c). One patient reported an improved WOMAC Pain score (CS1, 3 point improvement), exceeding the XLH-specific clinically meaningful threshold of a 2.2 point improvement (Skrinar et al., 2019b). One patient reported a decreased score (CS3, 2 point decrease); the score was not reported for CS2. The testimonials support these reported improvements in pain: ‘After taking burosumab, the pain began to ease’ (CS1) and ‘My general pain and energy levels are better’ (CS2). WOMAC Stiffness scores improved in all patients (by 4, 3, and 4 points), which greatly exceeds the XLH-specific clinically meaningful threshold of a 1 point improvement (Skrinar et al., 2019b). Patients also reported less stiffness: ‘I have so much … less stiffness’ (CS3).

Patients were more expansive when describing improvements in their mobility. ‘Three weeks after the first dose I was able to walk around my home unaided. Six weeks later I found myself walking down the street not needing to use my walking stick. Eight weeks later I felt confident to leave the house without any walking aids. Five months later I climbed some steps into the front of my house; I had been unable to do this for years. Friends and family have remarked how much improved my mobility is. My husband says he can see I don't need to cling on to the furniture to get around if I get up in the night, I just get up and go. He's amazed at the difference in me. I believe it has changed my life’ (CS1). ‘In the morning it doesn't take me as long to be able to get up and start moving around. I can put weight on my legs easier when I first get up whereas before it would take up to an hour before I could stand up and walk properly ... My knees and ankles feel so much stronger and more stable. I'm able to balance better when standing in one spot and when I'm walking’ (CS2). There were also descriptions of improvements in other areas of physical function: ‘Now I can crouch down and get back up without much difficulty … I feel much stronger overall, especially in my legs. … Now I can get up off the ground without even using my hands’ (CS2). ‘I am able to be more active, enjoying life … The improvements within my body are enormous, my friends and family have noticed the difference. I can manage a whole day out and not be over tired the next day’ (CS3).

The impact of burosumab treatment extends beyond the direct musculoskeletal effect, with patients also describing improvements in their energy levels and reduced fatigue. ‘My … energy levels are better. I feel more alert, less lethargic and less fatigued since starting my treatment…’ (CS2). ‘…I feel so alive. I have so much more energy, less fatigue…’ (CS3). They also reported benefits in mental health: ‘Since starting burosumab my mental health has improved also. The increase in energy and decreased fatigue has really helped me feel more motivated and positive in myself. Being in less pain and feeling stronger has benefited my mental wellbeing a lot’ (CS2); ‘… enjoying life not just physically but also mentally’ (CS3).

Improvements in HRQL index scores were dramatic: all index scores were above 0.7 at follow-up, increasing by 0.513, 0.604, and 0.021 points. This was reflected in improved EQ-VAS scores for two patients (35 and 50 point improvements for CS1 and CS2, respectively); CS3 had the same EQ-VAS score as at baseline (65). Patients also described improvements in their overall health: ‘I have not felt like this for a while because my overall health has been better since being treated with burosumab’ (CS2). ‘If burosumab was available years ago my life would have been so different … It was hard to explain about living with XLH as when you are born with a condition you just get on with your life, but I now know what it is sort of like to be just normal with having burosumab’ (CS3).

## 3 Discussion

The detriment described for these patients is consistent with published descriptions of XLH (Carpenter et al., 2011; Linglart et al., 2014). The musculoskeletal features are consistent with published reports and include rickets, joint deformity, spinal stenosis, osteoarthritis, and fractures requiring correction by orthopaedic surgery. The clinical presentation of CS2 was less typical in that they did not have any orthopaedic history. The patients also reported tinnitus and hearing loss, and poor dental health (abscesses and loss of teeth). Similar to published descriptions (Che et al., 2016; Cole et al., 2023; Javaid et al., 2022; Lo et al., 2020; Skrinar et al., 2019a), the three patients in this case series presented with pain, stiffness, fatigue, and low mood.

Laboratory and PRO evidence from the time before burosumab treatment and changes following burosumab treatment in this real-world setting reflect findings from clinical trials (Briot et al., 2021; Insogna et al., 2018; Kamenicky et al., 2023; Portale et al., 2019). Serum phosphate levels improved to normal or near-normal levels after 1 year of burosumab treatment and other biomarkers were maintained at normal levels or improved. PRO data showed clinically relevant improvements in pain, measured using the BPI-SF, and stiffness, measured using the WOMAC Index, both of which have been validated for use in XLH (Skrinar et al., 2019b; Skrinar et al., 2019c). The EQ-5D-5L index and EQ-VAS were not collected in the clinical trials but demonstrate improvements following treatment with burosumab in this case series.

In this case series, laboratory and PRO data are combined with patient testimonials to gain unique insight that could not have been obtained solely from the laboratory and PRO data. We learn about the extent of the disability before burosumab treatment in the patients' own words, including debilitating mobility problems, the need for physical support from others, difficulties performing daily activities, and tiredness and fatigue as a daily presence. The patients provided expansive descriptions of improvements in mobility whilst receiving burosumab, and of other improvements in physical function, improved energy levels, less fatigue, and improved mental wellbeing. The testimonials brought to life the quantitative PRO data in the patients' own words.

Our case series is limited by the availability of data for laboratory values during the time patients were receiving burosumab (when monitoring is often transferred out of secondary care to primary care), and inconsistencies in the time frame for the follow-up laboratory and PRO data. A case series of a small number of patients meeting the specific EAP inclusion criteria precludes generalization to the broader patient population, particularly noting that XLH is a heterogeneous disease with wide-ranging phenotypes and clinical manifestations (Beck-Nielsen et al., 2019). Whilst the BPI-SF and WOMAC Index have been validated for use in XLH, the EQ-5D-5L has not, although it is routinely used to capture HRQL data to support economic evaluations across numerous indications.

In summary, these three case studies provide support for the benefit of burosumab treatment in a real-world setting in adults with XLH who have persistent symptoms despite conventional therapy. The findings are consistent with published clinical trial data. Patient testimonials provide deeper insight into the burden of XLH and the benefits of treatment with burosumab. EAP data will shortly be reported, providing further evidence of the impact of burosumab treatment. In addition, the XLH Disease Monitoring Program (NCT03651505) is an international, prospective, 10-year, longitudinal, observational study of adults and children with XLH that will also contribute to this body of knowledge.

## CRediT authorship contribution statement

**Julia Day:** Conceptualization, Data curation, Funding acquisition, Investigation, Methodology, Writing – review & editing. **Chandrin Jayatilleke:** Conceptualization, Data curation, Investigation, Methodology, Writing – review & editing. **Matthew Roy:** Conceptualization, Data curation, Investigation, Methodology, Writing – review & editing.

## Declaration of competing interest

CJ and MR have received support for attending meetings and travel from Kyowa Kirin International (KKI); MR has received consulting fees from KKI.

## Data Availability

The authors do not have permission to share data.
